# Intrapulmonary Pharmacokinetics of Relebactam, a Novel β-Lactamase Inhibitor, Dosed in Combination with Imipenem-Cilastatin in Healthy Subjects

**DOI:** 10.1128/AAC.01411-17

**Published:** 2018-02-23

**Authors:** Matthew L. Rizk, Elizabeth G. Rhee, Patricia A. Jumes, Mark H. Gotfried, Tian Zhao, Eric Mangin, Sheng Bi, Cynthia M. Chavez-Eng, Zufei Zhang, Joan R. Butterton

**Affiliations:** a Merck & Co., Inc., Kenilworth, New Jersey, USA; b Pulmonary Associates, Phoenix, Arizona, USA

**Keywords:** relebactam, imipenem, intrapulmonary pharmacokinetics, healthy subjects

## Abstract

This phase I study assessed the intrapulmonary pharmacokinetic profiles of relebactam (MK-7655), a novel β-lactamase inhibitor, and imipenem. Sixteen healthy subjects received 250 mg relebactam with 500 mg imipenem-cilastatin, given intravenously every 6 h for 5 doses, and were randomized to bronchoscopy/bronchoalveolar lavage at 0.5, 1, 1.5, or 3 h after the last dose (4 subjects per time point). Both drugs penetrated the epithelial lining fluid (ELF) to a similar degree, with the profiles being similar in shape to the corresponding plasma profiles and with the apparent terminal half-lives in plasma and ELF being 1.2 and 1.3 h, respectively, for relebactam and 1.0 h in both compartments for imipenem. The exposure (area under the concentration-time curve from time zero to infinity) in ELF relative to that in plasma was 54% for relebactam and 55% for imipenem, after adjusting for protein binding. ELF penetration for relebactam was further analyzed by fitting the data to a two-compartment pharmacokinetic model to capture its behavior in plasma, with a partitioning coefficient capturing its behavior in the lung compartment. In this model, the time-invariant partition coefficient for relebactam was found to be 55%, based on free drug levels. These results support the clinical evaluation of relebactam with imipenem-cilastatin for the treatment of bacterial pneumonia.

## INTRODUCTION

Relebactam is a dual class A and class C β-lactamase inhibitor that can restore the *in vitro* activity of imipenem against many carbapenem-nonsusceptible isolates of Pseudomonas aeruginosa, Klebsiella pneumoniae, and Enterobacter spp. (1–4). The pharmacokinetic (PK) parameter best correlated with relebactam efficacy is the area under the concentration-time curve (AUC), with required exposures (AUC from time zero to 24 h [AUC_0–24_]) of ∼100 μM · h in a thigh infection model (5) and 150 μM · h in a pulmonary infection model (6). The increased exposures required in the pulmonary infection model are likely partially due to the penetration of relebactam into the murine lung, which is approximately 34%, based on the ratio of total drug levels in the lung to total drug levels in plasma (data on file). Relebactam doses of 125 mg or higher provide exposures (AUC from time zero to infinity [AUC_0–∞_]) that exceed the single-dose target of 37.5 μM · h, which is derived from the target of 150 μM · h after four times daily dosing established in the pulmonary infection model (6). The pharmacokinetic half-life of relebactam is compatible with four-times-daily dosing with imipenem-cilastatin (7–9), and coadministration of relebactam with imipenem-cilastatin has been generally well tolerated in phase 2 clinical studies (10, 11).

Imipenem is an ideal partner for β-lactamase inhibitors in pseudomonads from a resistance perspective. Imipenem is a potent carbapenem antibiotic that is relatively stable to the AmpC class C cephalosporinase of P. aeruginosa, requiring the concomitant loss of the entry porin OprD along with the hyperproduction of AmpC before resistance is achieved (12, 13). Unlike the β-methyl carbapenems, imipenem is not subject to efflux by any of the resistance-nodulation-cell division (RND)-type efflux pumps of Pseudomonas, including MexAB/OprM, MexCD/OprJ, MexEF/OprN, and MexXY/OprM (14, 15). Therefore, inhibition of the chromosomal enzyme by a β-lactamase inhibitor restores susceptibility to many multidrug-resistant isolates of P. aeruginosa, including those with overexpression of efflux pumps, but does not restore susceptibility in isolates where a β-lactamase not inhibited by relebactam is present, such as class B metallo-β-lactamases (16).

The penetration of antibiotics into tissues and fluids at the specific site of infection is a potentially valuable indicator for predicting a clinical response (17). For bacterial pneumonia, the distal bronchial lumen and alveolar surface are considered the sites of bacterial invasion (18, 19). Antibiotic concentrations in epithelial lining fluid (ELF) remain the most critical parameter for activity against extracellular pathogens, including most Gram-negative bacteria. The recovery of ELF by fiberoptic bronchoscopy and bronchoalveolar lavage (BAL) is a safe, well-tolerated procedure that has become widely used and accepted to study pulmonary drug penetration (20).

In this study, we assessed the pharmacokinetic profiles of relebactam and imipenem in the pulmonary ELF and alveolar cells (AC) obtained from BAL fluid specimens. Relebactam lung penetration was further analyzed by fitting the data to a two-compartment pharmacokinetic model to capture its behavior in the plasma, with a partitioning coefficient capturing its behavior in the lung compartment.

## RESULTS

Seventeen subjects (14 males, 3 females; age range, 24 to 42 years) entered the study. Sixteen subjects completed the study; one subject discontinued early due to an adverse event (see below).

**FIG 1:**
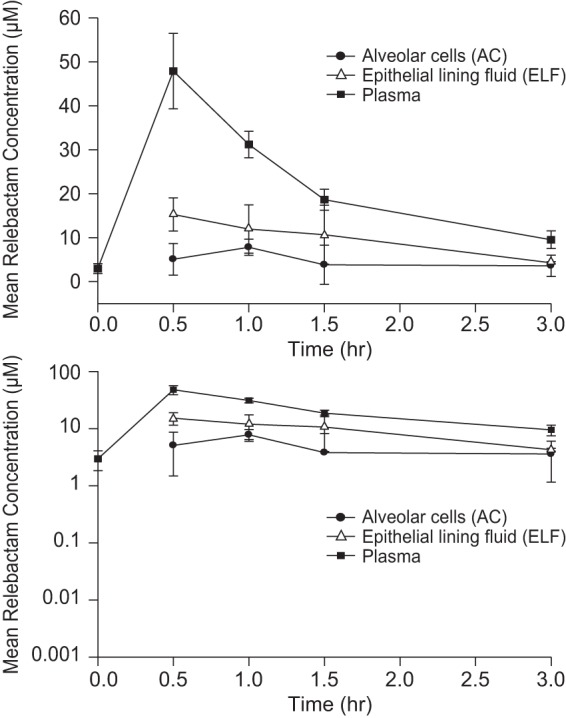
Arithmetic mean (±SD) concentration profiles for relebactam in plasma, ELF, and AC after multiple-dose administration of relebactam at 250 mg with imipenem-cilastatin at 500 mg in healthy subjects (*n* = 4 subjects per time point). (Top) Linear scale; (bottom) semilog scale.

Following 5 consecutive doses of relebactam at 250 mg in combination with imipenem-cilastatin at 500 mg given every 6 h, the relebactam levels in ELF and AC were consistently lower than the relebactam levels in plasma (Fig. 1). The penetration of relebactam into the extracellular space was approximately one-third to one-half of the corresponding level of penetration into plasma, with the geometric mean ratios (GMRs) for ELF/plasma concentrations ranging from 0.32 to 0.51 across time points (Table 1). The relative exposure (AUC_0–∞_) of relebactam in ELF versus plasma was 54% on the basis of the mean profiles (Table 2), after adjustment for protein binding (relebactam is 80% unbound in plasma; a free fraction of 100% was assumed for ELF). The time to the maximum concentration (*T*_max_) and the terminal half-life values for relebactam in ELF were similar to those in plasma, with *T*_max_ occurring at 0.5 h in both matrices and terminal half-lives being 1.2 h in plasma and 1.3 h in ELF (Table 2), indicating a lack of any system hysteresis.

**TABLE 1:** GMR for intrapulmonary concentration to plasma concentration of relebactam and imipenem after multiple-dose administration of relebactam at 250 mg with imipenem-cilastatin at 500 mg in healthy subjects^*a*^

Time (h)	Relebactam		Imipenem ELF/plasma concn GMR (90% CI)
	ELF/plasma concn GMR (90% CI)	AC/plasma concn GMR (90% CI)	
0.5	0.32 (0.23, 0.43)	0.14 (0.10, 0.19)	0.32 (0.25, 0.43)
1.0	0.35 (0.26, 0.47)	0.25 (0.18, 0.33)	0.36 (0.27, 0.48)
1.5	0.51 (0.38, 0.69)	0.38 (0.26, 0.56)	0.55 (0.42, 0.73)
3.0	0.46 (0.34, 0.62)	0.51 (0.36, 0.70)	0.50 (0.38, 0.67)

a Data are for four subjects at each time point. GMR, geometric mean ratio, which is the ratio of the least-squares means from the linear mixed-effect model performed on the natural log-transformed values with location, time (4 levels), and the location-by-time interaction as fixed effects and subject as a random effect. CI, confidence interval.

**TABLE 2:** PK parameters for relebactam and imipenem after multiple-dose administration of relebactam at 250 mg with imipenem-cilastatin at 500 mg in healthy subjects^*e*^

Analyte	Matrix	AUC_0–∞_ (μM · h)^*a*^	AUC_0–3_ (μM · h)^*a*^	*C*_max_ (μM)^*a*^	*T*_max_ (h)^*a*^	*t*_1/2_ (hr)^*a*^	ELF/plasma AUC_0–∞_ ratio^*c*^	Adjusted ELF/plasma AUC_0–∞_ ratio^*d*^
Relebactam							43.0	53.7
	Plasma	81.2	64.7	47.9	0.50	1.24		
	ELF	34.9	26.7	15.3	0.50	1.29		
	AC	23.6	12.8	7.81	1.00	2.25		
Imipenem							44.2	55.2
	Plasma	130	114	99.6	0.50	0.95		
	ELF	57.4	48.4	32.6	0.50	1.03		
	AC	—^*b*^	—	—	—	—		

a Concentration values were averaged across 4 subjects at each time point, and data for all time points were combined into a single data set for noncompartmental analysis calculation.

b —, insufficient data were available.

c Calculated as 100 · ELF AUC_0–∞_/plasma AUC_0–∞_.

d Calculated as 100 · ELF AUC_0–∞_/plasma AUC_0–∞_/0.8 (80% fraction unbound for both relebactam and imipenem).

e Data are for four subjects at each time point. Noncompartmental analysis was conducted on the mean profile. *C*_max_, maximum concentration; *t*_1/2_, terminal half-life.

The penetration of relebactam into the intracellular space was lower than that into ELF, with the GMRs for AC/plasma concentrations ranging from 0.14 to 0.51 across time points (Table 1) and the relative exposure (AUC_0–∞_) in AC versus plasma being 36% on the basis of the mean profiles after adjustment for protein binding. The *T*_max_ and terminal half-life for relebactam in AC were slightly different from those in plasma, representing slower intracellular penetration and clearance for AC than for plasma, with *T*_max_ occurring at 1.0 h and the terminal half-life being 2.3 h in AC.

**FIG 2:**
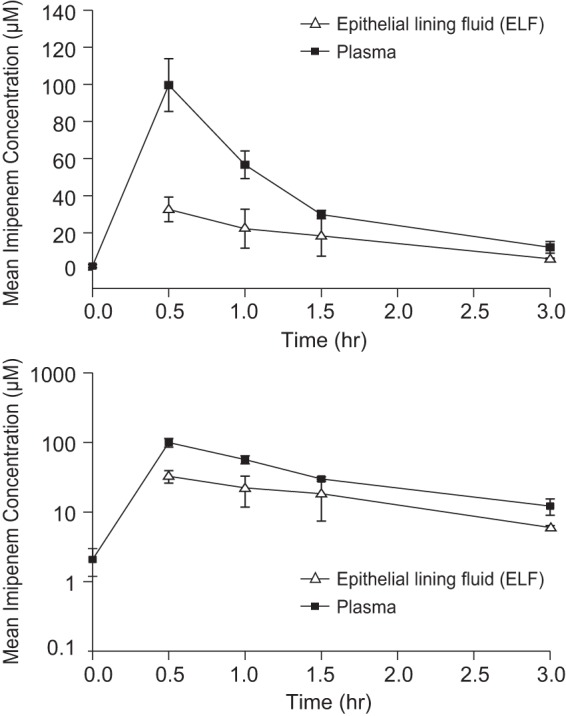
Arithmetic mean (±SD) concentration profiles for imipenem in plasma and ELF after multiple-dose administration of relebactam at 250 mg with imipenem-cilastatin at 500 mg in healthy subjects (*n* = 4 subjects per time point). (Top) Linear scale; (bottom) semilog scale.

As shown in Fig. 2, the imipenem levels in ELF were consistently lower than the imipenem levels in plasma. Because the large majority of imipenem AC concentrations were below the limit of quantitation, pharmacokinetic parameters for imipenem in AC are not reported. The penetration of imipenem into the extracellular space was approximately one-third to one-half of the corresponding level of penetration into plasma, with GMRs for ELF/plasma concentrations ranging from 0.32 to 0.55 across time points (Table 1) and the relative exposure (AUC_0–∞_) in ELF versus plasma being 55% on the basis of the mean profiles (Table 2), after adjustment for protein binding (imipenem is 80% unbound in plasma; a free fraction of 100% was assumed for the ELF). The *T*_max_ and terminal half-life for imipenem in ELF were similar to those in plasma, with *T*_max_ occurring at 0.5 h and the terminal half-life being 1.0 h in both matrices (Table 2), again indicating a lack of any observable system hysteresis.

**FIG 3:**
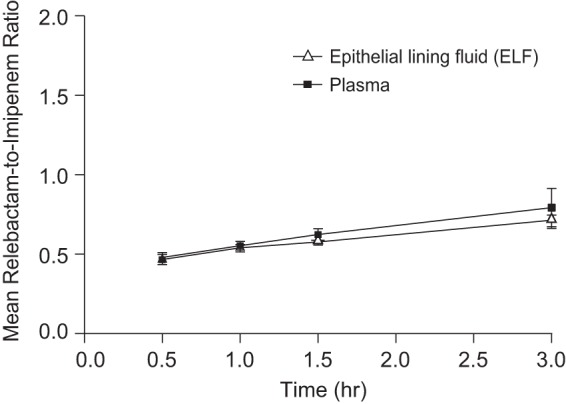
Arithmetic mean ratios (±SD) of relebactam to imipenem concentrations in plasma and ELF after multiple-dose administration of relebactam at 250 mg with imipenem-cilastatin at 500 mg in healthy subjects (*n* = 4 subjects per time point).

Mean relebactam concentration-to-imipenem concentration ratios in plasma and ELF are shown in Fig. 3. The primary hypothesis that the relebactam concentration in ELF would be >25% of the imipenem concentration in ELF at the *T*_max_ of imipenem (0.5 h) was confirmed, as the point estimate of the GMR for the relebactam concentration versus the imipenem concentration in ELF was 47% (90% confidence interval [CI], 45%, 49%) (Table 3).

**TABLE 3:** Relebactam and imipenem concentrations in ELF after multiple-dose administration of relebactam at 250 mg with imipenem-cilastatin at 500 mg in healthy subjects^*a*^

Time (h)	Relebactam concn in ELF (μM)		Imipenem concn in ELF (μM)		Relebactam/imipenem concn ratio	
	GM^*b*^	95% CI^*c*^	GM	95% CI	GMR^*d*^	90% CI
0.5	14.93	9.89, 22.53	32.09	21.26, 48.44	0.47	0.45, 0.49
1	10.93	7.24, 16.50	20.27	13.43, 30.59	0.54	0.52, 0.56
1.5	9.49	6.29, 14.32	16.47	10.92, 24.87	0.58	0.55, 0.60
3	4.27	2.83, 6.45	5.99	3.97, 9.04	0.71	0.68, 0.74

a Data are for four subjects at each time point.

b GM, geometric mean, which is the back-transformed least-squares mean from the linear mixed-effects model performed on the natural log-transformed values with for compound (MK and IPM), time (4 levels), and the compound-by-time interaction as fixed effects and subjects as a random effect.

c CI, confidence interval.

d GMR, geometric mean ratio.

**FIG 4:**
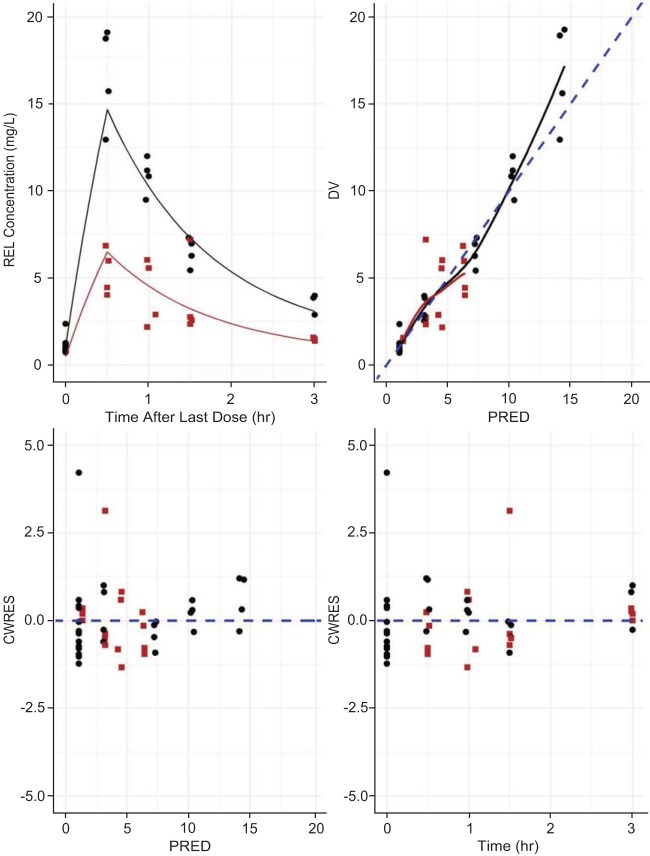
Relebactam population PK model diagnostics (DV, directly observed value; PRED, model-predicted value; CWRES, conditionally weighted residual) for plasma (black circles) and ELF (red squares) data. REL, relebactam.

The population PK analysis of relebactam indicated that among the three approaches explored, the ELF data were best fit by a time-invariant partition coefficient driven by the predicted unbound plasma concentration. Model parameters are shown in Table 4, with the corresponding model diagnostics being presented in Fig. 4. As shown in Table 4, the partition coefficient estimated by the model (55%) was consistent with the estimated penetration obtained using the AUC ratio method described above (54%). The plasma and lung PK of relebactam were sufficiently described by the two-compartment plasma model, with a time-invariant partitioning describing penetration into the lung. The model predicts that the equilibration of relebactam between the plasma and ELF is rapidly established with negligible delay, with substantial penetration into the ELF occurring.

**TABLE 4:** Relebactam population PK model parameter estimates^*a*^

Model and parameter	Parameter abbreviation	Units	Estimated value	% RSE
Structural model				
Clearance	θ_CL_	liters/h	9.17	5.04
Volume of distribution in the central compartment	*θ_V_c__*	liters	15.3	15.9
Volume of distribution in the peripheral compartment	*θ_V_p__*	liters	10.6	384.9
Intercompartmental clearance	*θ_Q_2__*	liters/h	2.64	107.2
Residual error				
Additive	σ_addi_	mg/liter	0.01 (fixed)	
Proportional	σ_prop_	Percent CV	28.0	49.5
ELF penetration				
Penetration coefficient for ELF	*θ_k_ELF__*	Ratio	0.553	9.06
Proportional residual error	*σ*_prop,*k_ELF_*_	Percent CV	39.2	44.2

a RSE, relative standard error; CV, coefficient of variation.

Five subjects reported a total of 9 adverse events during the study; all were nonserious. Five events were deemed to be drug related: mild fatigue in one subject, a mild increase in the creatinine concentration in another subject, and mild diarrhea with moderate nausea and vomiting in a third subject. The subject with nausea, vomiting, and diarrhea was discontinued from the study during administration of the second dose of study drug. All adverse events resolved, and most events (7 of 9) lasted less than 12 h.

## DISCUSSION

In this study, the penetration of both relebactam and imipenem into the pulmonary extracellular space was similar, with relative exposures in ELF versus plasma being 54% for relebactam and 55% for imipenem, after adjustment for protein binding, on the basis of the ratio of the AUC values between the respective compartments. These values were confirmed using population PK approaches, where penetration into the ELF was similarly projected to be 55% for both relebactam and imipenem (21). The *T*_max_ for both compounds was 0.5 h in both matrices, and the terminal half-lives were also similar (1.2 h in plasma and 1.3 h in ELF for relebactam, 1.0 h in both matrices for imipenem), indicating the rapid establishment of equilibrium for both relebactam and imipenem between plasma and ELF and a lack of any significant delay or system hysteresis. These observations also lend support to the sampling scheme chosen for this study and the decision not to collect additional data at later time points at the end of the dosing interval, as the parallel elimination phases in plasma and ELF observed for both imipenem and relebactam indicated a robust characterization of the clearance in both compartments. The plasma PK parameters observed in this study for both relebactam and imipenem were consistent with those previously reported in healthy subjects (7, 8).

Penetration of relebactam into the intracellular space resulted in the relative exposure of relebactam in AC versus plasma of 36%, after adjustment for protein binding, and relebactam was cleared more slowly from AC than from plasma (half-lives, 2.3 versus 1.2 h). Imipenem levels in AC were undetectable in the majority of patients, consistent with the general observation that β-lactams do not penetrate into intracellular compartments as well as macrolides and fluoroquinolones (18). This finding is not clinically meaningful, since the efficacy of imipenem-cilastatin for the treatment of pneumonia has been well established in several large, multicenter clinical trials (22).

Although imipenem penetration into the respiratory tract has not been studied previously, extensive clinical experience in the treatment of pneumonia suggests that the exposures achieved in ELF are sufficient for clinical efficacy, even if they are lower than those observed in plasma. As detailed above, results from preclinical *in vivo* infection models indicate that the PK parameter best correlated with relebactam efficacy is the AUC, with exposures (AUC_0–24_) of ∼100 μM · h being required in the thigh infection model and exposures of 150 μM · h being required in the pulmonary infection model. The increased exposures required in the pulmonary infection model are likely partially due to the penetration of relebactam into the murine lung, which is approximately 34% on the basis of the ratio of total drug levels in the plasma versus total drug levels in the lung (data on file). Because the ELF/plasma concentration ratio for relebactam is slightly lower in mice than in humans, plasma concentrations represent a good surrogate for lung exposure. Thus, the plasma PK target from the mouse lung infection model allows a direct assessment of the appropriateness of dosing for lung infections, using the corresponding human plasma PK data. The plasma PK derived from phase 2 studies in patients have been previously analyzed and reported, indicating robust target attainment at the relebactam dose of 250 mg four times daily (10).

Prior studies of the intrapulmonary penetration of carbapenem-class β-lactam antibiotics are limited and have not included imipenem. Other carbapenems have shown penetration ratios that bracket the ELF/plasma concentration ratios observed for imipenem in this study. For example, the ELF/plasma concentration ratio for meropenem ranged from 0.32 to 0.53 after multiple-dose administration (1 g every 8 h for 4 doses) in healthy adults (23) and was estimated to be 30% on the basis of population modeling in patients with ventilator-associated pneumonia (24). For ertapenem, ratios of ELF concentrations to total plasma concentrations ranged from 0.21 to 0.64 (median, 0.32) in a multiple-dose study of adult patients with ventilator-associated pneumonia (25).

Prior studies of the intrapulmonary penetration of β-lactam–β-lactamase inhibitor combination therapies are also limited. For orally administered amoxicillin-clavulanate, concentrations of both drugs in ELF were less than 20% of those observed in plasma (26). In critically ill patients receiving multiple doses of piperacillin-tazobactam, mean concentrations in ELF were approximately 57% and 91% of the total concentrations in plasma for piperacillin and tazobactam, respectively (27). In healthy adults, ceftazidime-avibactam displayed similar plasma and ELF curves, with ELF/plasma AUC ratios being 31% to 32% for ceftazidime and 32% to 35% for avibactam (28). Ceftolozane-tazobactam has also demonstrated similar ELF and plasma curves, with ELF/plasma AUC ratios being 48% for ceftolozane and 44% for tazobactam in healthy adults (29). In a recent study of meropenem-RPX7009 in healthy adults, mean penetration ratios based on AUC were 63% for meropenem and 53% for RPX7009 (30).

The accuracy of antibiotic measurements in ELF and AC can be influenced by several methodological issues. In particular, the prolonged dwelling time of fluid during BAL (>1 min) can cause additional urea to diffuse into ELF and overestimate the ELF volume. Contamination of BAL fluid with blood can also lead to overestimation of the ELF volume and inaccurate drug concentration estimates. Since antibiotic concentrations may differ in fluids and cells, prompt separation of cells from fluid is necessary to avoid the lysis of cells, which may change the concentrations in fluid. To minimize the effects of these factors, this study was conducted by experienced personnel using established bronchoscopy and BAL procedures and included detailed procedures for the collection, handling, and storage of BAL fluid samples, with careful and prompt separation of ELF and AC. In addition, the concentrations at several time points spanning the dosing interval were studied, providing enough data to generate a pulmonary PK profile and to support a PK/pharmacodynamic hypothesis. The primary limitation of this study (as opposed to the general limitations of ELF studies) is that it was conducted in healthy volunteers, and there is limited information available regarding the correlation of pulmonary drug penetration in healthy volunteers to that in critically ill patients. Though they are limited, current data indicate that the lung penetration ratios observed in healthy volunteers appears to be directionally and often quantitatively similar to the penetration ratios observed in patients (31). Furthermore, the conduct of such a study in healthy subjects is common practice (32), due to feasibility considerations as well as the semiquantitative interpretability of study results (21). Further, while we observed parallel elimination slopes between the observed plasma and ELF data over the range of observed data, additional sampling through the full dosing interval of 6 h would provide an even more complete picture of the dynamic lung penetration of relebactam.

In summary, this study in healthy subjects demonstrates that relebactam and imipenem achieve similar relative exposures in pulmonary ELF and plasma and that the relebactam and imipenem clearances seen in pulmonary ELF mirror those seen in plasma. Imipenem was not detected in AC, providing further confirmation that the activity of imipenem in the pulmonary extracellular compartment (ELF) may be most relevant to its efficacy in treating pneumonia. These data suggest that a dose adjustment for either relebactam or imipenem is likely not necessary for the effective treatment of bacterial pneumonia. Relebactam is sufficiently well tolerated to continue with further clinical investigation, and these results further support the investigation of relebactam used in combination with imipenem-cilastatin in a phase 3 trial for the treatment of bacterial pneumonia (ClinicalTrials.gov registration no. NCT02493764).

## MATERIALS AND METHODS

This open-label, randomized, parallel-group study (MK-7655 protocol 007) was conducted from 24 April 2012 through 25 June 2012 at a single site (Pulmonary Associates, Phoenix, AZ) and was in conformance with principles of good clinical practice, as well as all applicable statutes or regulations regarding the protection of the rights and welfare of human subjects participating in biomedical research. The protocol was approved by the Quorum Review Institutional Review Board, and all subjects gave written informed consent before any study procedures were performed. The primary objective was to determine the relationship between the intrapulmonary pharmacokinetics of relebactam and those of imipenem after four-times-daily intravenous administration of relebactam with imipenem-cilastatin in healthy subjects. The primary study hypothesis was that the relebactam concentration in ELF would be >25% of the imipenem concentration in ELF at the *T*_max_ of imipenem (0.5 h).

Eligible subjects were healthy men and women 18 to 45 years of age with a body mass index of ≤32 kg/m^2^, a creatinine clearance of ≥80 ml/min, no clinically significant disease, and no history of significant multiple or severe allergies, including allergies to β-lactam antibiotics and lidocaine or other local anesthetics. The subjects received 5 doses of study drug (relebactam at 250 mg in combination with imipenem-cilastatin at 500 mg) by intravenous infusion over 30 min, with one dose being administered every 6 h, and subsequently underwent bronchoscopy and BAL at either 0.5, 1.0, 1.5, or 3.0 h after the last dose of study drug. Dosing and administration of imipenem-cilastatin were carried out in a manner consistent with the recommended labeled posology.

For each subject, the timing of bronchoscopy/BAL was determined by random assignment to panel A, B, C, or D (4 subjects per panel). An optional fifth panel (panel E) was included to allow the collection of information at a different dose or time point (such as at the end of the dosing interval at 6 h), contingent upon the analysis of the intrapulmonary and plasma PK data from panels A to D. On the basis of the data obtained for panels A to D, it was decided not to conduct an analysis with panel E, as the data from panels A to D were deemed adequate by visual inspection to characterize the intrapulmonary profiles of imipenem and relebactam.

BAL fluid specimens were obtained during bronchoscopy for the determination of the relebactam and imipenem concentrations in the ELF and AC. Four aliquots of normal saline (50 ml each) were sequentially instilled and aspirated after each instillation. The first aspirate was discarded, and the remaining aspirates were pooled and used to obtain urea and drug level measurements. Aliquots were obtained for cell count/differential, and the remainder of the pooled aspirates was centrifuged. The liquid aspirate and cell pellet were separated and stored at −70°C. The supernatant was sent for urea and drug level measurements. Blood samples were collected prior to the first and fifth doses of study drug for the determination of relebactam and imipenem plasma concentrations. Blood samples were also collected during the bronchoscopy/BAL procedure for the determination of relebactam, imipenem, and urea concentrations.

The safety and tolerability of relebactam were monitored by clinical assessment of adverse events, measurement of vital signs, and performance of a physical examination, 12-lead electrocardiogram (ECG), and standard laboratory safety tests (hematology, chemistry, and urinalysis). Renal function (established by determination of serum/urine creatinine, serum urea, urine protein, and urine albumin concentrations) and hepatic function (established by determination of serum bilirubin, alanine aminotransferase, and aspartate aminotransferase concentrations) were carefully monitored during the study. The safety of bronchoscopy and BAL was monitored by clinical assessment, including continuous cardiac monitoring and repeated measurements of vital signs, according to the standard operating procedures at the study site.

### Analytical and pharmacokinetic methods.

Relebactam and imipenem levels in plasma, pulmonary ELF, and AC were measured simultaneously via acetonitrile protein precipitation and hydrophilic interaction liquid chromatography (HILIC) with detection via liquid chromatography-tandem mass spectrometry (LC-MS/MS). The system consisted of a Waters Acquity ultraperformance liquid chromatograph (Waters Corp., Milford, MA) and an API 4000 or 5000 triple-quadrupole tandem mass spectrometer (Sciex, Framingham, MA) equipped with a turbo-ion spray interface and operated in the positive ionization mode. The multiple reaction monitoring (MRM) transitions monitored were *m/z* 349 → 269 for relebactam, *m/*z 300 → 98 for imipenem, and *m/z* 354 → 274 and *m/z* 307 → 98 for their respective internal standards. The chromatographic separation of the analytes was achieved using a Waters Atlantis HILIC (50 by 2.1 mm by 3 μm) column kept at 35°C and a mobile phase consisting of 5 mM ammonium acetate (pH 4.5) in 80:20 acetonitrile-water. For the plasma assay, the flow rates and run times were 0.45 ml/min and 3.0 min, respectively; for the ELF and AC assays, the flow rate and run time were 0.4 ml/min and 4.0 min, respectively.

The ELF volumes recovered by BAL were determined by using urea as an endogenous marker to provide a dilution ratio by measurement of urea concentrations in the BAL fluid and serum (33). The concentrations in AC were determined by estimation of the intracellular volume of macrophages (i.e., 2.42 ml per 10^6^ cells), on the basis of the cell count/differential. The mean value for each time point was used to conduct the noncompartmental analysis using Phoenix (version 6.3) software (Pharsight Corporation, Mountain View, CA) and to calculate the values of the PK parameters on the basis of the mean profile in each matrix.

Population PK analysis was conducted using NONMEM (version 7.3) software (ICON plc., Dublin, Ireland). The first-order conditional estimation with interaction (FOCEI) method was applied for parameter estimation. A previous population PK analysis showed that the plasma concentration-time profile of relebactam can be sufficiently described by a two-compartment model with linear PK (10). To elucidate the relationship between plasma and ELF concentrations, penetration into the ELF was explored using three approaches, each using a naive pooled data approach: (i) a three-compartment model with bidirectional mass transfer between the ELF compartment and the central volume, (ii) a time-invariant partition coefficient driven by the predicted unbound plasma concentration, and (iii) an effect compartment with an input rate constant driven by the concentration difference between the volume in the central compartment and the effect compartment.

The equations comprising the partition coefficient model are as follows:

(1) $$\frac{{dA}_{1}}{dt}=\frac{Q_{2}}{V_{2}}A_{2}-\frac{Q_{2}}{V_{1}}A_{1}-\frac{\text{CL}}{V_{1}}A_{1}$$

(2) $$\frac{{dA}_{2}}{dt}=\frac{Q_{2}}{V_{1}}A_{1}-\frac{Q_{2}}{V_{2}}A_{2}$$

(3) $$C_{\text{ELF}}=k_{\text{ELF}}\cdot f_{u,\text{REL}}\cdot \frac{A_{1}}{V_{1}}$$

where *A*_1_ is the amount of drug in the central compartment, *A*_2_ is the amount of drug in the peripheral compartment, CL is the plasma clearance, *Q*_2_ is the intercompartmental clearance, *V*_1_ is the volume of distribution in the central compartment, *V*_2_ is the volume of distribution in the peripheral compartment, *C*_ELF_ is the relebactam concentration in the ELF, *f_u_*_,ELF_ is the free fraction of relebactam in plasma (∼80%), *k*_ELF_ is the partition coefficient for distribution into the ELF space, and *t* is time.

The free fraction in the ELF was assumed to be 100%. Different residual error models (additive, proportional, and combined) were tested for both the plasma and ELF concentrations of relebactam. Model development, including the selection of the structural and residual error model, was based upon the success of minimization, numerical comparison of the objective function values, the precision of the parameter estimates, and the generation of standard model diagnostic plots. The PK of imipenem were not modeled, as the lung penetration data from this study were previously described using a population PK approach (21).

### Statistical analysis.

All data were analyzed according to the treatment actually received. Safety and tolerability were assessed in the all-subjects-as-treated (AST) population, defined as all subjects who received at least one dose of study drug. Pharmacokinetic parameters were analyzed in the per-protocol (PP) population, defined as subjects who complied sufficiently with the protocol to ensure that the data would likely exhibit the effects of treatment, according to the underlying scientific model.

The concentrations of relebactam and imipenem in pulmonary ELF were log transformed and analyzed using a linear mixed-effects model containing compound (relebactam and imipenem), time (30 min, 1 h, 1.5 h, and 3 h after the last dose), and the compound-by-time interaction as fixed effects and subject as a random effect. Point estimates and 90% confidence intervals (CI) were calculated for the geometric mean ratio (GMR) of the concentration (relebactam concentration/imipenem concentration) at the time to the maximum concentration (*T*_max_) of imipenem on the basis of the mean concentration-time profile. The log-trapezoidal rule was used to compute the AUC_0–3_ of relebactam and imipenem in the ELF, AC, and plasma for the mean concentration-time profile.

## References

[1] Livermore DM, Warner M, Mushtaq S 2013 Activity of MK-7655 combined with imipenem against Enterobacteriaceae and Pseudomonas aeruginosa. J Antimicrob Chemother 68:2286–2290. doi:10.1093/jac/dkt178. 23696619

[2] Hirsch EB, Ledesma KR, Chang KT, Schwartz MS, Motyl MR, Tam VH 2012 In vitro activity of MK-7655, a novel beta-lactamase inhibitor, in combination with imipenem against carbapenem-resistant Gram-negative bacteria. Antimicrob Agents Chemother 56:3753–3757. doi:10.1128/AAC.05927-11. 22526311 PMC3393460

[3] Lob SH, Hackel MA, Kazmierczak KM, Hoban DJ, Young K, Motyl MR, Karlowsky JA, Sahm DF 2017 In vitro activity of imipenem-relebactam against gram-negative bacilli isolated from patients with lower respiratory tract infections in the United States in 2015—results from the SMART Global Surveillance Program. Diagn Microbiol Infect Dis 88:171–176. doi:10.1016/j.diagmicrobio.2017.02.018. 28291628

[4] Lob SH, Hackel MA, Kazmierczak KM, Young K, Motyl MR, Karlowsky JA, Sahm DF 2017 In vitro activity of imipenem-relebactam against Gram-negative ESKAPE pathogens isolated by clinical laboratories in the United States in 2015 (results from the SMART Global Surveillance Program). Antimicrob Agents Chemother 61:2209–2216. doi:10.1128/AAC.02209-16. PMC5444184 28320716

[5] Mavridou E, Melchers RJ, van Mil AC, Mangin E, Motyl MR, Mouton JW 2015 Pharmacodynamics of imipenem in combination with beta-lactamase inhibitor MK7655 in a murine thigh model. Antimicrob Agents Chemother 59:790–795. doi:10.1128/AAC.03706-14. 25403667 PMC4335905

[6] Powles MA, Galgoci A, Misura A, Liberator P, Hammond ML 2010 In vivo efficacy of the beta-lactamase inhibitor, MK-7655, in combination with imipenem in murine models of infection, abstr F1-2140. Abstr 50th Intersci Conf Antimicrob Agents Chemother. American Society for Microbiology, Washington, DC.

[7] Butterton JR, Jumes P, Calder N, Rizk ML, Nefliu M, Sun P, Schwartz M, Mangin E, Warrington W, Stoch A, Wagner JA 2010 A phase I study evaluating the safety, tolerability, and pharmacokinetics of an intravenous beta-lactamase inhibitor in healthy male volunteers, abstr F1-1967. Abstr 50th Intersci Conf Antimicrob Agents Chemother. American Society for Microbiology, Washington, DC.

[8] Jumes P, Rizk ML, Calder N, Gutierrez M, Warrington S, Li X, Liu Y, Stoch A, Wagner JA, Butterton JR 2012 Phase I studies evaluating the safety, tolerability, and pharmacokinetics of multiple doses of an intravenous beta-lactamase inhibitor in healthy young males and single doses in healthy elderly male, elderly female and young female volunteers, abstr A-009. Abstr 52nd Intersci Conf Antimicrob Agents Chemother. American Society for Microbiology, Washington, DC.

[9] Rizk ML, Jumes P, Lasseter K, Marbury T, Mangin E, Liu Y, Wagner J, Butterton J 2012 Pharmacokinetics of MK-7655, a novel β-lactamase inhibitor (bli), in combination with imipenem/cilastatin (IPM/CIL) in subjects with impaired renal function, abstr A-010. Abstr 52nd Intersci Conf Antimicrob Agents Chemother. American Society for Microbiology, Washington, DC.

[10] Lucasti C, Vasile L, Sandesc D, Venskutonis D, McLeroth P, Lala M, Rizk ML, Brown ML, Losada MC, Pedley A, Kartsonis NA, Paschke A 2016 Phase 2, dose-ranging study of relebactam with imipenem-cilastatin in subjects with complicated intra-abdominal infection. Antimicrob Agents Chemother 60:6234–6243. doi:10.1128/AAC.00633-16. 27503659 PMC5038313

[11] Sims M, Mariyanovski V, McLeroth P, Akers W, Lee YC, Brown ML, Du J, Pedley A, Kartsonis NA, Paschke A 2017 Prospective, randomized, double-blind, phase 2 dose-ranging study comparing efficacy and safety of imipenem/cilastatin plus relebactam with imipenem/cilastatin alone in patients with complicated urinary tract infections. J Antimicrob Chemother 72:2616–2626. doi:10.1093/jac/dkx139. 28575389

[12] Lister PD, Wolter DJ, Hanson ND 2009 Antibacterial-resistant Pseudomonas aeruginosa: clinical impact and complex regulation of chromosomally encoded resistance mechanisms. Clin Microbiol Rev 22:582–610. doi:10.1128/CMR.00040-09. 19822890 PMC2772362

[13] Livermore DM 1992 Interplay of impermeability and chromosomal beta-lactamase activity in imipenem-resistant Pseudomonas aeruginosa. Antimicrob Agents Chemother 36:2046–2048. doi:10.1128/AAC.36.9.2046. 1329641 PMC192435

[14] Okamoto K, Gotoh N, Nishino T 2002 Alterations of susceptibility of Pseudomonas aeruginosa by overproduction of multidrug efflux systems, MexAB-OprM, MexCD-OprJ, and MexXY/OprM to carbapenems: substrate specificities of the efflux systems. J Infect Chemother 8:371–373. doi:10.1007/s10156-002-0193-7. 12525903

[15] Masuda N, Sakagawa E, Ohya S, Gotoh N, Tsujimoto H, Nishino T 2000 Substrate specificities of MexAB-OprM, MexCD-OprJ, and MexXY-oprM efflux pumps in Pseudomonas aeruginosa. Antimicrob Agents Chemother 44:3322–3327. doi:10.1128/AAC.44.12.3322-3327.2000. 11083635 PMC90200

[16] Blizzard TA, Chen H, Kim S, Wu J, Bodner R, Gude C, Imbriglio J, Young K, Park YW, Ogawa A, Raghoobar S, Hairston N, Painter RE, Wisniewski D, Scapin G, Fitzgerald P, Sharma N, Lu J, Ha S, Hermes J, Hammond ML 2014 Discovery of MK-7655, a beta-lactamase inhibitor for combination with Primaxin(R). Bioorg Med Chem Lett 24:780–785. doi:10.1016/j.bmcl.2013.12.101. 24433862

[17] Rizk ML, Zou L, Savic RM, Dooley KE 2017 Importance of drug pharmacokinetics at the site of action. Clin Transl Sci 10:133–142. doi:10.1111/cts.12448. 28160433 PMC5421734

[18] Stein GE, Wells EM 2010 The importance of tissue penetration in achieving successful antimicrobial treatment of nosocomial pneumonia and complicated skin and soft-tissue infections caused by methicillin-resistant Staphylococcus aureus: vancomycin and linezolid. Curr Med Res Opin 26:571–588. doi:10.1185/03007990903512057. 20055750

[19] Baldwin DR, Honeybourne D, Wise R 1992 Pulmonary disposition of antimicrobial agents: in vivo observations and clinical relevance. Antimicrob Agents Chemother 36:1176–1180. doi:10.1128/AAC.36.6.1176. 1416817 PMC190300

[20] Rodvold KA, Yoo L, George JM 2011 Penetration of anti-infective agents into pulmonary epithelial lining fluid: focus on antifungal, antitubercular and miscellaneous anti-infective agents. Clin Pharmacokinet 50:689–704. doi:10.2165/11592900-000000000-00000. 21973267

[21] Van Hasselt JG, Rizk ML, Lala M, Chavez-Eng Visser CSA, Kerbusch T, Danhof M, Rao G, Van der Graaf PH 2016 Pooled population pharmacokinetic model of imipenem in plasma and the lung epithelial lining fluid. Br J Clin Pharmacol 81:1113–1123. doi:10.1111/bcp.12901. 26852277 PMC4876184

[22] Rodloff AC, Goldstein EJ, Torres A 2006 Two decades of imipenem therapy. J Antimicrob Chemother 58:916–929. doi:10.1093/jac/dkl354. 16997845

[23] Conte JE Jr, Golden JA, Kelley MG, Zurlinden E 2005 Intrapulmonary pharmacokinetics and pharmacodynamics of meropenem. Int J Antimicrob Agents 26:449–456. doi:10.1016/j.ijantimicag.2005.08.015. 16280244

[24] Lodise TP, Sorgel F, Melnick D, Mason B, Kinzig M, Drusano GL 2011 Penetration of meropenem into epithelial lining fluid of patients with ventilator-associated pneumonia. Antimicrob Agents Chemother 55:1606–1610. doi:10.1128/AAC.01330-10. 21300830 PMC3067164

[25] Boselli E, Breilh D, Saux MC, Gordien JB, Allaouchiche B 2006 Pharmacokinetics and lung concentrations of ertapenem in patients with ventilator-associated pneumonia. Intensive Care Med 32:2059–2062. doi:10.1007/s00134-006-0401-5. 17039351

[26] Cook PJ, Andrews JM, Woodcock J, Wise R, Honeybourne D 1994 Concentration of amoxycillin and clavulanate in lung compartments in adults without pulmonary infection. Thorax 49:1134–1138. doi:10.1136/thx.49.11.1134. 7831630 PMC475276

[27] Boselli E, Breilh D, Cannesson M, Xuereb F, Rimmele T, Chassard D, Saux MC, Allaouchiche B 2004 Steady-state plasma and intrapulmonary concentrations of piperacillin/tazobactam 4 g/0.5 g administered to critically ill patients with severe nosocomial pneumonia. Intensive Care Med 30:976–979. doi:10.1007/s00134-004-2222-8. 15057512

[28] Nicolau DP, Siew L, Armstrong J, Li J, Edeki T, Learoyd M, Das S 2015 Phase 1 study assessing the steady-state concentration of ceftazidime and avibactam in plasma and epithelial lining fluid following two dosing regimens. J Antimicrob Chemother 70:2862–2869. doi:10.1093/jac/dkv170. 26133566

[29] Chandorkar G, Huntington JA, Gotfried MH, Rodvold KA, Umeh O 2012 Intrapulmonary penetration of ceftolozane/tazobactam and piperacillin/tazobactam in healthy adult subjects. J Antimicrob Chemother 67:2463–2469. doi:10.1093/jac/dks246. 22773741

[30] Wenzler E, Gotfried MH, Loutit JS, Durso S, Griffith DC, Dudley MN, Rodvold KA 2015 Meropenem-RPX7009 concentrations in plasma, epithelial lining fluid, and alveolar macrophages of healthy adult subjects. Antimicrob Agents Chemother 59:7232–7239. doi:10.1128/AAC.01713-15. 26349830 PMC4649232

[31] Valitalo PA, Griffioen K, Rizk ML, Visser SA, Danhof M, Rao G, van der Graaf PH, van Hasselt JG 2016 Structure-based prediction of anti-infective drug concentrations in the human lung epithelial lining fluid. Pharm Res 33:856–867. doi:10.1007/s11095-015-1832-x. 26626793

[32] European Medicines Agency. 2015 Guideline on the use of pharmacokinetics and pharmacodynamics in the development of antibacterial medicinal products. European Medicines Agency, London, United Kingdom http://www.ema.europa.eu/docs/en_GB/document_library/Scientific_guideline/2015/09/WC500194333.pdf.

[33] Rennard SI, Basset G, Lecossier D, O'Donnell KM, Pinkston P, Martin PG, Crystal RG 1986 Estimation of volume of epithelial lining fluid recovered by lavage using urea as marker of dilution. J Appl Physiol (1985) 60:532–538. doi:10.1152/jappl.1986.60.2.532. 3512509

